# Reanalysis of whole-exome sequencing (WES) data of children with neurodevelopmental disorders in a standard patient care context

**DOI:** 10.1007/s00431-023-05279-4

**Published:** 2023-10-27

**Authors:** Michelle van Slobbe, Arie van Haeringen, Lisenka E. L. M. Vissers, Emilia K. Bijlsma, Julie W. Rutten, Manon Suerink, Esther A. R. Nibbeling, Claudia A. L. Ruivenkamp, Saskia Koene

**Affiliations:** 1 Department of Clinical Genetics, Leiden University Medical Centre, Leiden, The Netherlands; 2 Department of Human Genetics, Donders Centre for Neuroscience, Radboud University Medical Center, Nijmegen, The Netherlands

**Keywords:** Whole-exome sequencing, Reanalysis, Diagnosis, Intellectual disability, Neurodevelopmental disorder

## Abstract

This study aims to inform future genetic reanalysis management by evaluating the yield of whole-exome sequencing (WES) reanalysis in standard patient care in the Netherlands. Single-center data of 159 patients with a neurodevelopmental disorder (NDD), in which WES analysis and reanalysis were performed between January 1, 2014, and December 31, 2021, was retrospectively collected. Patients were included if they were under the age of 18 years at initial analysis and if this initial analysis did not result in a diagnosis. Demographic, phenotypic, and genotypic characteristics of patients were collected and analyzed. The primary outcomes of our study were (i) diagnostic yield at reanalysis, (ii) reasons for detecting a new possibly causal variant at reanalysis, (iii) unsolicited findings, and (iv) factors associated with positive result of reanalysis. In addition, we conducted a questionnaire study amongst the 7 genetic department in the Netherlands creating an overview of used techniques, yield, and organization of WES reanalysis. The single-center data show that in most cases, WES reanalysis was initiated by the clinical geneticist (65%) or treating physician (30%). The mean time between initial WES analysis and reanalysis was 3.7 years. A new (likely) pathogenic variant or VUS with a clear link to the phenotype was found in 20 initially negative cases, resulting in a diagnostic yield of 12.6%. In 75% of these patients, the diagnosis had clinical consequences, as for example, a screening plan for associated signs and symptoms could be devised. Most (32%) of the (likely) causal variants identified at WES reanalysis were discovered due to a newly described gene-disease association. In addition to the 12.6% diagnostic yield based on new diagnoses, reclassification of a variant of uncertain significance found at initial analysis led to a definite diagnosis in three patients. Diagnostic yield was higher in patients with dysmorphic features compared to patients without clear dysmorphic features (yield 27% vs. 6%; *p* = 0.001).

*Conclusions*: Our results show that WES reanalysis in patients with NDD in standard patient care leads to a substantial increase in genetic diagnoses. In the majority of newly diagnosed patients, the diagnosis had clinical consequences. Knowledge about the clinical impact of WES reanalysis, clinical characteristics associated with higher yield, and the yield per year after a negative WES in larger clinical cohorts is warranted to inform guidelines for genetic reanalysis. These guidelines will be of great value for pediatricians, pediatric rehabilitation specialists, and pediatric neurologists in daily care of patients with NDD.

**Table:** 

**What is Known:** *• Whole exome sequencing can cost-effectively identify a genetic cause of intellectual disability in about 30–40% of patients.* *• WES reanalysis in a research setting can lead to a definitive diagnosis in 10–20% of previously exome negative cases.*	
**What is New:** *• WES reanalysis in standard patient care resulted in a diagnostic yield of 13% in previously exome negative children with NDD.* *• The presence of dysmorphic features is associated with an increased diagnostic yield of WES reanalysis.*

**Supplementary Information:**

The online version contains supplementary material available at 10.1007/s00431-023-05279-4.

## Introduction

In Western countries, intellectual disability (ID), with a global prevalence of 1–8% [1–3], is one of the principal socio-economic healthcare problems [3] and is among the conditions with the highest healthcare costs [4]. In Europe, the prevalence of the more broadly defined neurodevelopmental disorders (NDD) is estimated to be around 5–10% [5, 6]. Pathogenic genetic variants are estimated to cause up to 40% of the cases with NDD [7, 8]. Finding a cause for NDD is of great importance to both the patient and the family, providing insight into the prognosis and recurrence risks as well as possible treatment options for some cases [9]. Whole-exome sequencing (WES) is the currently most used technique to screen for pathogenic genetic variants [10]. Knowledge about the clinical impact of WES reanalysis and clinical characteristics associated with higher yield and yield per year after a negative WES in larger clinical cohorts is warranted to inform guidelines for genetic reanalysis. These guidelines will be of great value for pediatricians, pediatric rehabilitation specialists, and pediatric neurologists in daily care of patients with NDD.

Although many diagnoses are made using WES, with a diagnostic yield of around 28% in specific ID cohorts and 36% in cohorts of children with neurodevelopmental delay, many patients remain undiagnosed [11, 12]. Possible explanations are missing the causative variant in the regular exome sequencing pipeline (intronic variants, low coverage, filtering/quality issues) or detection of a variant in a gene not (yet) associated with disease [13, 14]. As variant detection techniques are constantly improving and each year 250 new gene-disease interactions and 9200 new variant-disease associations are described in literature [9], repeating exome analysis after some time can increase diagnostic yield [9, 15, 16].

The yield of WES reanalysis has been studied in multiple research cohorts with varying phenotypes, resulting in yields ranging between 6 and 47% [9, 13, 15–31]. A recent systematic review in patients with suspected Mendelian disorders showed an overall diagnostic yield of WES reanalysis of 10% (95% CI 6–13%) [32]. In cohorts with mostly NDD patients, the observed diagnostic yield of reanalysis is between 11 and 18% in larger studies (50 or more reanalyzed cases) and 29% and 36% in two small studies of 14 patients each [9, 13, 17, 20, 22, 29]. Although these studies in research populations indicate that systematic reanalysis of data can improve the diagnostic yield in patients with NDD, these studies do not provide sufficient insight in the benefits of WES reanalysis in standard patient care. A study in clinical patient care, consisting for more than half of patients with NDD, in 2017 showed that WES reanalysis of single patient data after 8–17 months yielded no new diagnoses [33].

Since regular WES reanalysis in all undiagnosed patients with NDD is expected to be associated with high healthcare costs, more information is required on characteristics associated with a high(er) yield. In a research setting, (cost-)effectiveness rises with the increase of interval between analyses [34] and selecting patient groups with a higher chance of a positive result [35].

To gain insight into these parameters as well as the yield of WES reanalysis in standard patient care, we studied a cohort of children with NDD at the Leiden University Medical Centre (LUMC).

## Methods

### Data collection

**Fig. 1:**
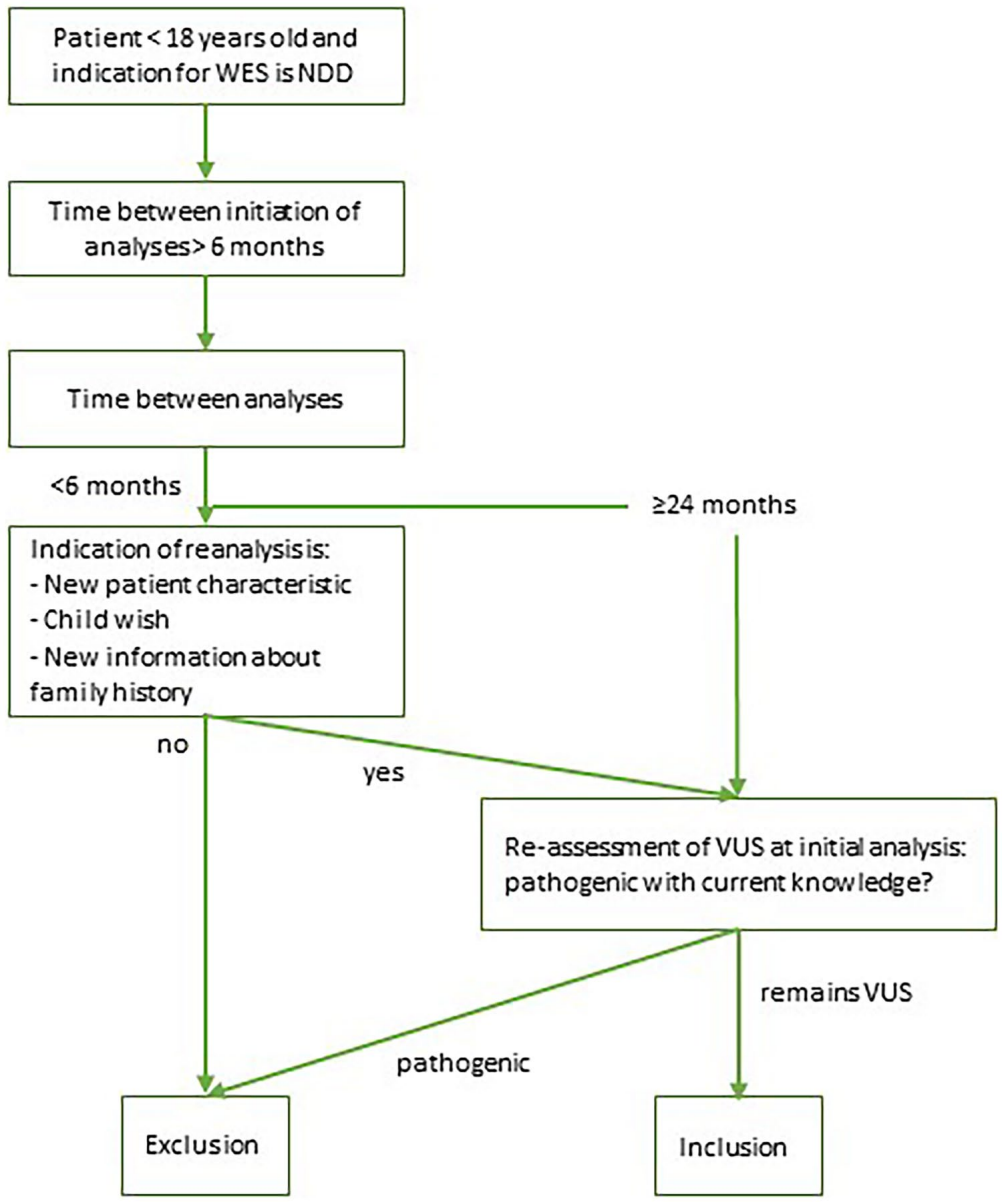
Inclusion criteria. Flow diagram displaying criteria for inclusion

We collected data of children with NDD in whom WES analysis and reanalysis were performed between January 1, 2014, and December 31, 2021, in standard patient care in the LUMC. Patients were eligible for reanalysis if initial analysis had not resulted in a diagnosis. Before WES reanalysis was initiated, previously identified variants (variants of uncertain significance (VUS)) were first reevaluated (JWR, AH, SK, MS, EKB). If considered (likely) pathogenic, the patient was not included in this study. If the variant was still considered to be a VUS, the patient was included in the study. A second analysis was considered a reanalysis if the two analyses were performed more than two years apart, or if they were performed more than 6 months apart, but there was a specific reason for reanalysis (see Fig. 1). Patients were excluded if they were older than 18 years at initial analysis or if they did not have ID or NDD.

Demographic information, phenotypic characteristics, the presence of dysmorphic features, and genetic test specifications and outcomes were retrospectively obtained from chart review. Whether consanguinity existed was determined by anamnesis and was defined as a known common ancestor. All data were collected in an online database (CastorEDC).

To gain insight into the organization and yield of WES (re)analysis in the Netherlands, a questionnaire study was conducted among all clinical genetics departments of the academical medical centers in the Netherlands (*n* = 7) (for complete questionnaire translated to English, see Supplement material S1).

### Variant analysis

WES-based analyses were used for all patients in the cohort (for test characteristics per patient, see Supplementary material 3). WES reanalysis was performed on existing data; no new capture was performed. At the moment of the initial diagnostic request, exome sequencing was performed on an Illumina platform after exome enrichment with the Agilent SureSelectXT Human All Exon (V5 or V7) or SureSelect Clinical Research Exome V2 kits at Genomescan B.V., Leiden, the Netherlands. Burrows-Wheeler Aligner was used for read alignment, and Genome Analysis Tool Kit was used for variant calling. For the annotation of the variants, a specific in-house developed program was used. From 2018, data analysis was performed using Moon software, Diploid, Belgium**.** Based on patient sex, age of onset of symptoms, Human Phenotype Ontology (HPO) terms, and sequence data, Moon software prioritizes the variants using artificial intelligence [36]. In most cases, gene panel analysis (showing variants associated with known intellectual disability genes) was performed, followed by open exome analysis (showing variants in all genes, mostly used to search for de novo variants in genes not (yet) included in the gene panels) in some cases.

The laboratory reported variants of uncertain significance (VUS) in candidate genes (genes not yet associated with NDD but having a function that may be involved in the development of NDD), VUS in known genes, likely pathogenic variants and pathogenic variants in known genes. The latter two categories were merged in this study and referred to as (likely) pathogenic variants.

### Data analysis

The primary outcomes were (i) diagnostic yield at reanalysis, (ii) reasons for detecting a new possibly causal variant at reanalysis, (iii) unsolicited findings (clinically relevant findings not associated with the indication of the test), and (iv) factors associated with positive result of reanalysis. The diagnostic yield was defined as the percentage of cases for whom a (likely) pathogenic variant or VUS in a known gene with a clear link to the phenotype was identified. Factors tested for association with positive reanalysis were phenotypic and test characteristics, individual HPO terms, and disease groups based on HPO terms.

Normally distributed data were expressed by the mean and standard deviation, while skewed data were described by the median and range. To statistically compare groups, the Mann–Whitney test was conducted for continuous values and the two-sided Fisher exact test to compare the proportions within categorical variables. A *p*-value < 0.05 was considered statistically significant, and Bonferroni’s correction was used to correct for multiple testing [37]. All analyses were conducted using IBM SPSS Statistics version 25.

## Results

### Demographics

One hundred and fifty-nine patients were included in the LUMC: 63 females and 96 males (Table 1). Median age of the patients at the time of the initial analysis was 7 years (range 1 day–17 years). All had NDD, 54.4% of the patients had ID. Twenty-five patients were offspring of consanguineous parents (15.7%). A change in phenotypic characteristics between the first and second analyses was reported in 31.4% of the children.

**Table 1:** Characteristics and phenotypic features of the patients

**Characteristics**	**Patients (*n*** **= 159)**
**Gender**	
Female	63 (39.6%)
Male	96 (60.4%)
**Ethnicity**	
Caucasian	78 (49.1%)
Middle east and North African	36 (22.6%)
Middle and South African	7 (4.4%)
Asian	4 (2.5%)
Other	6 (3.8%)
Unknown	28 (17.6%)
**Age groups (years)**	
0–5	66 (41.5%)
6–11	64 (40.3%)
12–18	29 (18.2%)
**Consanguinity**	
Yes	25 (15.7%)
No	134 (84.3%)
**Intellectual disability**	
Yes	86 (54.1%)
No	44 (27.7%)
Unknown	29 (18.2%)
**Total IQ score (*n* = 71) ± SD**	64 ± 14
**Neurological disorder**	
Yes	79 (49.7%)
* - Epilepsy*	*39 (24.5%)*
* - Hypotonia*	*26 (164%)*
* - Paresis*	*8 (5.0%)*
No	75 (47.2%)
Unknown	5 (3.1%)
**Autism spectrum disorder**	
Yes	63 (39.6%)
No	80 (50.3%)
Unknown	16 (10.1%)
**Dysmorphic features**	
Yes	44 (27.7%)
No	105 (66.0%)
Unknown	10 (6.3%)
**Brain abnormality on imaging**	
Yes	38 (23.9%)
No	114 (71.7%)
Unknown	7 (4.4%)
**Change in phenotype since initial analysis**	
Yes	50 (31.4%)
No	91 (57.2%)
Unknown	18 (11.3%)

### Initial genetic data analysis and reanalysis

The initial analyses were conducted with gene panel followed by open analysis of the complete exome as the most used strategy (67%). For the reanalyses, an HPO-based analysis in combination with a complete exome analysis was most frequently used (76%; Supplementary material S2). The mean time between the analyses was 3.7 years (range 0.5–8.4 years). In most cases, the reanalysis was initiated by the clinical geneticist (65%) or treating physician (29%; mainly pediatricians, pediatric neurologists, general practitioners, intellectual disability physicians). Parents initiated the reanalysis in 6% of cases. In 13.8% of patients, a new analysis was initiated due to the development of a new phenotypic characteristic.

### HPO terms

HPO terms were registered for the patients in which HPO-based analysis was performed at reanalysis (*n* = 154). In total, 795 terms were used, with a median of 5.2 HPO terms per patient (range 1–19 terms). Of the 312 unique terms that were used to describe the patients, “HP:0000750 Delayed speech and language development,” “HP:0000717 Autism” and “HP:0001256 Intellectual disability, mild” were used most (Supplementary material S4).

### Diagnostic yield of reanalysis

**Fig. 2:**
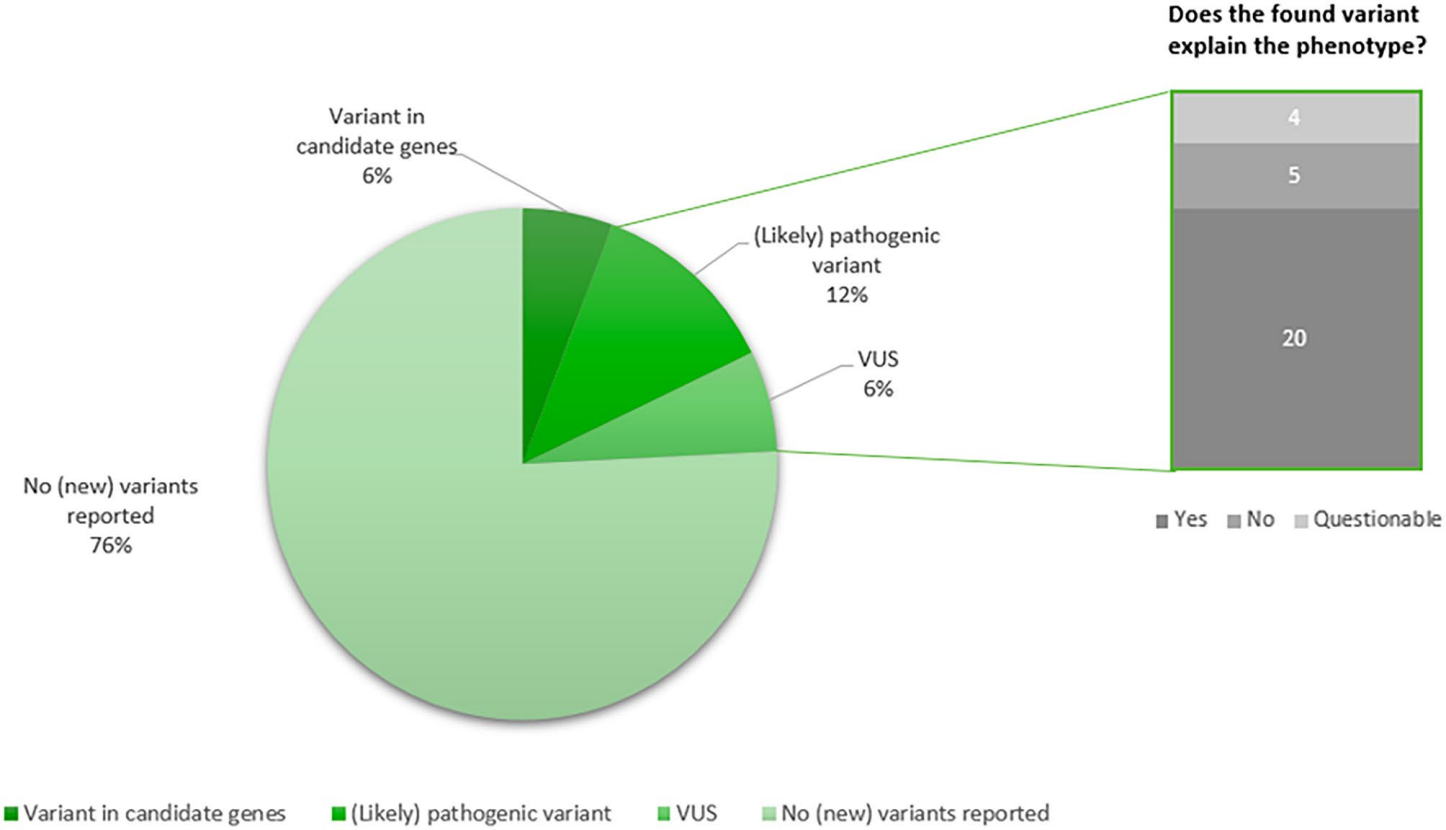
New variants found at reanalysis. (Left) Distribution of types of new variants discovered by reanalysis. (Right) Further specification of clinical significance of found VUS and (likely) pathogenic variants

In thirty-eight patients (23.9%), a new variant was reported at reanalysis (Fig. 2). There were no unsolicited findings. A (likely) pathogenic variant in a known gene, or VUS with a clear link to the phenotype, was found in twenty cases (12.6%; Table 2). These diagnoses influenced the further treatment policy in 15 (75.0%) of these patients (family planning advice, screening plan for associated signs and symptoms, referral to a Centre of Expertise).

**Table 2:** Explanatory variants found at reanalysis

**Patient ID**	**Gene**	**OMIM number**	**Mode of inheritance**	**Variant type**	**Zygosity**	**Inheritance**	**Classification**	**Why found**	**Treatment policy effect**
7	*FOXP1*	*605515	AD	Nonsense	Heterozygous	De novo	(Likely) pathogenic	Filtering update	Screening-related diseases
24	*FOXP1*	*605515	AD	Splice-site	Heterozygous	Paternal mosaicism	(Likely) pathogenic	Filtering update	Screening-related diseases
26	*TSC2*	*191092	AD	Missense	Heterozygous	Maternal	VUS in known gene	Moon Analysis	Referral to specialized center Screening-related diseases
33	*BPTF*	*601819	AD	Frameshift	Heterozygous	N/A	(Likely) pathogenic	New gene discovery	Screening-related diseases Possibly diagnosing siblings
35	*FBXO11*	*607871	AD	Splice-site	Heterozygous	De novo	(Likely) pathogenic	New gene discovery	Screening-related diseases
39	*GRIN2B*	*138252	AD	Missense	Heterozygous	De novo	(Likely) pathogenic	More knowledge about gene	Screening-related diseases
51	*OTUD6B*	*612021	AR	Missense	Homozygous	De novo	VUS in known gene	New gene discovery	-
91	*KCNMA1*	*600150	AD/AR	Missense	Heterozygous	De novo	(Likely) pathogenic	New gene discovery	Referral to specialized center
93	*SETD5*	*615743	AD	Splice-site	Heterozygous	De novo	(Likely) pathogenic	Filtering update	Screening-related diseases
94	*CWC27*	*617170	AR	Frameshift	Homozygous	Biparental	(Likely) pathogenic	New gene discovery	-
114	*SPEN*	*613484	AD	Frameshift	Heterozygous	De novo	(Likely) pathogenic	New gene discovery	Screening-related diseases
121	*TAOK1*	*610266	AD	Nonsense	Heterozygous	De novo	(Likely) pathogenic	New gene discovery	Referral to specialized center
137	*PTCH1*	*601309	AD	In-frame deletion	Mosaic	N/A	(Likely) pathogenic	Change patient characteristics	Screening-related diseases
139	*AFF4*	*604417	AD	Missense	Heterozygous	Maternal mosaicism	(Likely) pathogenic	Filtering update	-
147	*SUZ12*	*6006245	AD	Splice-site	Heterozygous	De novo	(Likely) pathogenic	Gene panel update	Screening-related diseases
151	*SYT1*	*185605	AD	Missense	Heterozygous	De novo	(Likely) pathogenic	Gene panel update	Screening-related diseases
164	*TNRC6B*	*610740	AD	Frameshift	Heterozygous	Paternal	VUS in known gene	New gene discovery	Family planning information
171	*SPEN*	*613484	AD	Frameshift	Heterozygous	De novo	(Likely) pathogenic	New gene discovery	-
177	*POU3F3*	*602480	AD	Frameshift	Heterozygous	De novo	(Likely) pathogenic	New gene discovery	-
180	*SATB2*	*608148	AD	Nonsense	Heterozygous	De novo	(Likely) pathogenic	Gene panel update	Screening-related diseases

*GP, gene panel; MA, Moon analysis; OE, open exome analysis

Segregation information is not available if trio WES was not performed

Inheritance not known if pathogenicity of variant is not clear

In 20 patients, a conclusive diagnosis was found (Table 2). A likely pathogenic variant that was clearly linked to the phenotype was found in 17 patients. In patient 7 and 24, a (likely) pathogenic variant in the *FOXP1* gene was found due to an update in filtering. In patients 114 and 171, a (likely) pathogenic variant was found in the *SPEN* gene, of which the first gene-disease association was described in 2020, and therefore, it was not found at previous analyses. In the other patients, a variety of new (likely) pathogenic variants was found, mostly because of the discovery of new genes. A VUS was likely causal for the phenotype in three patients, based on of functional data (*n* = 1), and/or a clear phenotypic match. In patient 51, a homozygous missense variant was found in the *OTUD6B* gene. The VUS was not described previously in the medical literature, was observed only twice in control populations, was located in an evolutionarily conserved amino acid, and was predicted as pathogenic by multiple prediction programs. The phenotype of the patient matched the disease specification associated with the gene. In patient 26, a missense variant was found in the *TSC2* gene. This variant was predicted as pathogenic by multiple prediction programs, was never found in control population, and was located in an evolutionarily highly conserved amino acid. TSC function and expression were reduced. This variant fitted the characteristics of the patient, who had developmental delay despite an almost normal IQ, psychiatric problems, and epilepsy. In patient 164, a frameshift variant in the *TNRC6B* gene was found which lead to the early introduction of a termination codon. This VUS was never found in control populations and had never been described in literature. The patient had mild ID and behavioral problems, which fit the associated characteristics of the gene.

VUS or (likely) pathogenic variants with a questionable link to the phenotype were found in four cases (2.5%). In patient 140, a likely pathogenic nonsense variant in the *ZMYM2* gene was found (Supplementary material S3), for which the patient did not completely match the phenotype. In patient 5, a VUS (homozygous missense variant) in the *DCHS1* gene was found, of which the patient matched the developmental delay and hearing problems associated with the gene, but not the pronounced dysmorphisms. In patient 27, a maternally inherited VUS (heterozygous splice-site variant) in the *AGO1* gene was found. No inherited variants have been described before and the phenotype only matched the patient’s developmental problems. In patient 37, a (heterozygous in-frame-deletion) VUS in the *SIN3A* gene was found, which could be linked to the patient’s ID and behavioral problems; however, the patient had tall stature and no dysmorphisms associated with Witteveen-Kolk syndrome. Segregation analysis of the variant was not possible.

In five cases (3.1%), a VUS/(likely) pathogenic variant was found that did not explain the phenotype. In patient 75, a paternal VUS was discovered in the *RAD21* gene. She did not have any phenotypical characteristics associated with the gene apart from ID and her father was healthy. A VUS in *ASH1L*, found in patient 30, was also present in a healthy sister. In patient 6, a normal functional metabolic test made the X-linked inherited variant in *TMLHE* less likely causal. In patient 115, a paternal variant in *NPRL3* was found. In patient 72, the variant in the *IRF2BPL* gene was less likely to be pathogenic because of a mismatch with the described phenotype (severe neurological problems in early childhood, lacking at 19 years of age).

Reanalysis led to the detection of variants in candidate genes in nine patients (5.7%; Fig. 2; Supplementary material S3).

**Fig. 3:**
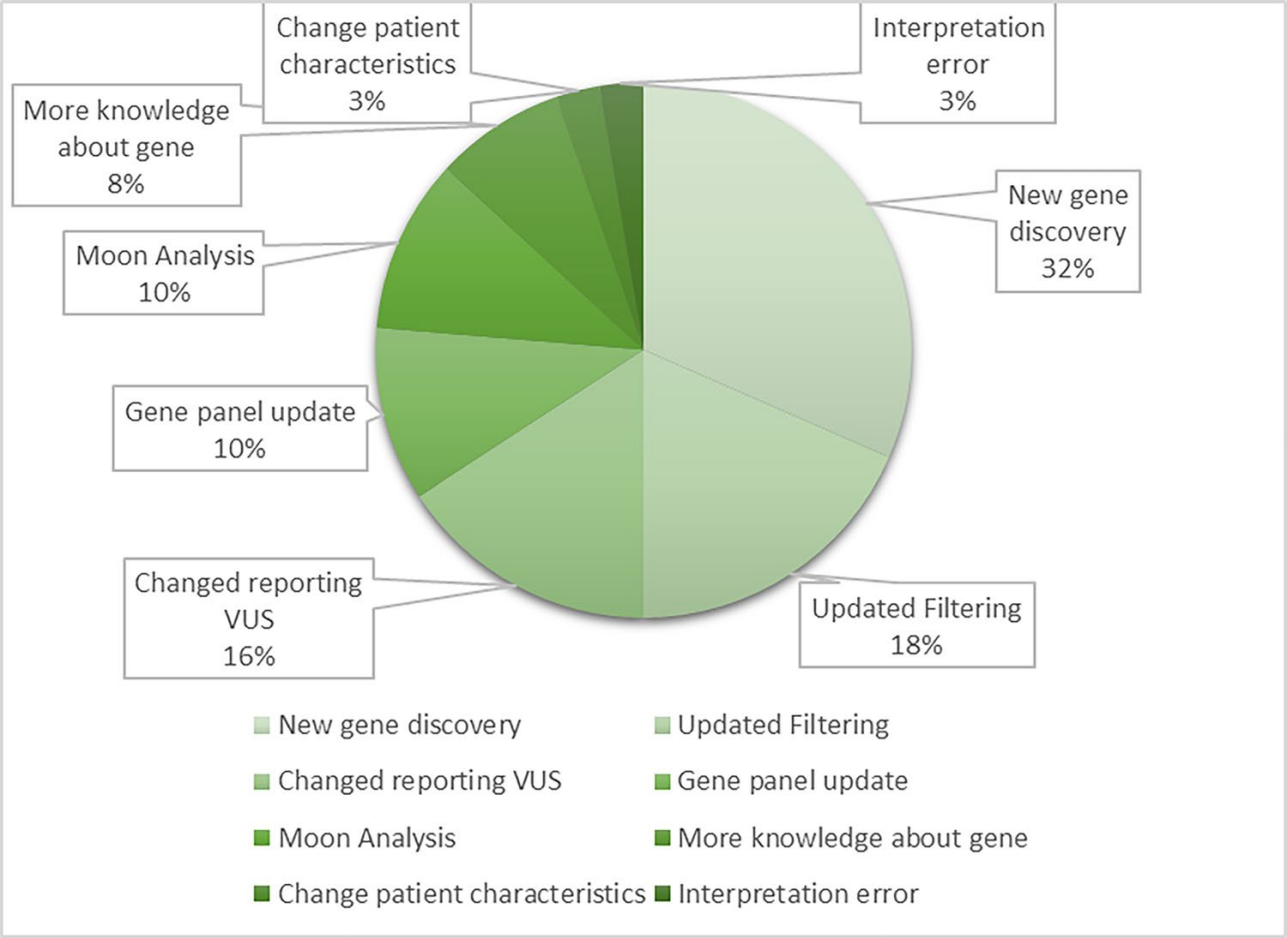
Reasons for discovering the variant *n* = 38 at reanalysis. The number of variants that is found for a specific reason is displayed on the *Y*-axis. Reasons for finding new variants at reanalysis were analyzed in collaboration with a laboratory specialist and grouped into the following categories: “New gene discovery”; “Updated filtering”; Changed reporting VUS”; “Gene panel update”; Moon analysis”; More knowledge about gene”; Change patient characteristics”; “Interpretation error.” If the gene-disease association of a particular variant had been discovered at an initial analysis but had not been included in the gene panel yet, the reason for finding the variant was categorized as “Gene panel update” at the time of reanalysis. If the gene-disease association was discovered after the initial analysis, the category “New gene discovery” was used. The identification of a new variant was categorized as “Moon analysis” if Moon analysis facilitated the identification, for example, by identifying a paternal/maternal variant as a possible diagnosis. If a change in patient characteristics resulted in diagnosis, the category “Change patient characteristics” was used. Changes in the filtering could also lead to an earlier missed variant, hence the category “Updated filtering”. If a variant was missed at initial analysis because it was mistakenly interpreted by the lab specialist, it was categorized as “Interpretation error.” Some VUS were found, but not reported at initial analysis due to the reporting guidelines at that time, hence the category “Changed reporting VUS.” If the knowledge about a gene-disease association expanded leading to a new diagnosis, the category “More knowledge about gene” was used

The largest proportion of new variants (12/38; 32%) was found due to recently published gene-disease associations (Fig. 3). Other major reasons for discovering new variants were updates in filtering (7/38; 18%), changes in VUS reporting (6/38; 16%), gene panel updates (4/38; 11%), and data analysis by Moon software (4/38; 11%).

### Reinterpretation of VUS

Fifty-two patients had a VUS in a known (*n* = 29) or candidate (*n* = 23) gene at the initial analysis. By reevaluating the phenotype and the existing knowledge and reanalysis, the VUS at the initial analysis was concluded to be more likely causal in three cases (5.8%). One VUS was concluded to be causal after the discovery of ataxia at the reevaluation by the neurologist and discussions with expert-colleagues in a young patient with a de novo missense variant in *SCN8A* who grew into the phenotype. One patient was included in a case series after reanalysis (de novo heterozygous missense variant in *GRIK2*), finding a similar phenotype in other patients with missense variants in this gene. The last patient with global developmental delay, cataract, and MRI abnormalities had a de novo missense variant in the *ITSN1*-gene, which was concluded to be likely causal because of the presence of the specific characteristic of cataract both in the patient and in other patients with variants in the guanine nucleotide exchange factor genes.

Including these three diagnoses, the total diagnostic yield is 14.5%.

The diagnostic yield in the group of patients who had VUS at first analysis was 19.2% vs. 12.1% for the patients in whom no VUS was detected at the previous analysis (*p* = 0.2). In six patients, the VUS was classified as probably benign after update.

### Predictors of positive reanalysis

We found a significantly higher diagnostic yield at reanalysis in patients with dysmorphic features compared to patients without dysmorphic features (*p* = 0.001; Table 3). No other clinical characteristics were significantly associated with diagnostic yield at reanalysis (Supplementary materials S4 and S5). There was a positive trend of microcephaly (*p* = 0.7) and abnormal muscle tone (*p* = 0.1); a negative trend was seen between autism and diagnostic yield (*p* = 0.3). The use of more than five HPO terms or an increased time between analyses was not associated with diagnostic yield.

**Table 3:** Diagnostic yield by demographic and phenotypic characteristics

**Characteristic**	***n*** = *	**Number of patients with characteristic**	**Diagnostic yield, %**		*p*-value**
			***Within with characteristic***	***Within without characteristic***	
Female gender	159	63	20.6	7.3	0.026
Initiator reanalysis parents/treating physician	159	56	14.3	11.7	0.626
Time interval > 5 years	159	13	15.4	12.3	0.669
Change in phenotype since initial analysis	141	50	8.0	14.3	0.418
Consanguinity	159	25	12.0	12.7	1
Intellectual disability	130	96	12.8	18.2	0.439
TIQ ≤ 55	71	22	13.6	12.4	1
Disharmonic intelligence profile	36	24	25.0	8.3	0.384
Autism spectrum disorder	143	63	9.5	16.3	0.323
Attention deficit hyperactivity disorder	143	19	5.3	14.5	0.469
Neurological disorder	154	79	11.4	12.0	1
* - Epilepsy*	157	40	7.5	13.7	0.405
Brain abnormality on imaging	152	38	0.0	15.8	0.007
Congenital anomalies					
* - Heart defects*	159	22	18.2	11.7	0.485
* - Congenital limb defect*	159	9	11.1	12.7	1
*- Cleft lip/palate*	158	1	0.0	12.7	1
Dysmorphic features	149	44	27.3	5.7	**0.001**

*If it was not clear whether a patient possessed a certain clinical feature, this was coded as a missing value and omitted from the analysis

**p-value by Fisher exact test for the comparison of the group with and without the specific characteristic. After Bonferroni’s correction *p*-value < 0.003 was considered statistically significant

Significant values are highlighted in bold

In twenty-two patients, reanalysis was initiated because a new phenotypical feature was observed in the patient. In this group, the time between the two analyses was on average 1.5 years shorter than in the group that had reanalysis on another indication (*p* < 0.01). The diagnostic yield was 4.5% (1/22) in this group, compared to 13.9% (19/137) for patients without new clinical features (*p* = 0.3).

### WES reanalysis policy in the Netherlands

The six other Dutch academic centers that were approached all participated in the study, and all performed WES reanalysis. In most centers, reanalysis of the data was performed after an initially negative WES, with a time interval depending on personal preference of the physician, phenotype severity, and age of the patient. Systemic reanalysis, defined as reanalysis of all previously exome negative patients, was not conducted in any center as part of routine care; two centers indicated this was mainly due to lack of capacity. Participants were generally convinced of the benefits of reanalysis in clinical practice but had concerns about the implementation. The stated concerns were related to the workload for clinical geneticists and clinical laboratory geneticists, the legal and psychological boundaries for automated reanalysis and healthcare costs.

One of the respondents, the Radboud University Medical Center, evaluated yield of reanalysis in the clinical setting. Reanalysis was performed on 329 children with neurodevelopmental disorders in whom no conclusive diagnosis was identified at initial analysis. This analysis now yielded a conclusive diagnosis in 8% (*n* = 26). In 16, this diagnosis was obtained due to identification of new variants, whereas in the other 10, this was based on reclassification of a previously identified VUS. In addition to new conclusive diagnoses, a possible diagnosis was obtained in 37% (*n* = 122 individuals, of which 49 were newly uncovered).

## Discussion

This study of reanalysis of WES data in standard patient care in children with NDD had a diagnostic yield of 12.6%. The diagnostic yield in this study in daily care in a single Dutch academic hospital largely corresponds to the diagnostic yield in previously performed larger studies [9, 13, 17, 22, 32]. Consistent with previous research, the main reason for detecting a new diagnosis at WES reanalysis was the discovery of new gene-disease associations [16, 32, 38]. The genetic diagnosis had medical implications in 75% of the cases with a definitive genetic diagnosis.

VUS reclassification led to a diagnosis in three cases. This is only a minor proportion of the yield compared to a recent study, in which VUS reclassification was the main reason for finding a new diagnosis [38]. Since the current study was part of standard patient care and previously detected VUS were evaluated before the initiation of WES reanalysis and patients with (likely) causal variants were not included in this study, the reported diagnostic yield could be an underestimate of the real diagnostic yield. Also, this difference could be explained by a difference in VUS reporting guidelines of the laboratory at initial diagnosis.

Patient selection based on characteristics associated with a higher diagnostic yield has been shown to increase initial diagnostic efficiency in a large machine-learning study [35]. In our study, only the presence of dysmorphic features was significantly associated with diagnostic yield. In the study by Dingemans et al., autism was negatively associated with diagnostic yield, while microcephaly and abnormal muscle tone were positively associated with diagnostic yield [35]. Interestingly, we found similar, although non-significant, associations between autism, microcephaly, and abnormal muscle tone and diagnostic yield in our cohort. These associations should be explored further in larger cohorts to determine their value in predicting a diagnostic genetic test result.

Guidelines describing how WES reanalysis should be organized in daily practice are required to keep genetic testing available for patients with NDD. Selection of patients on the presence of specific characteristics, e.g., dysmorphic features, microcephaly, or abnormal muscle tone, or rather absence (autism), may improve the cost-effectiveness of WES reanalysis, but stringent selection will inevitably lead to underdiagnosis. Earlier studies by Schobers et al. and the Solve-RD project studied the effect of doing a systematic reanalysis in combination with ad hoc analysis and found that this increased yield by 0.6–22% [38, 39]. Therefore, we could advocate for systematic reanalysis of WES-data in all undiagnosed patients; however, there are still many judicial, practical, healthcare system, and technical difficulties to overcome before successful implementation [38]. Finally, the role of the treating pediatricians, pediatric rehabilitation specialists, and pediatric neurologists in reanalysis management has to be clearly defined to facilitate identification of undiagnosed patients with a persistent suspicion of a genetic cause of disease.

## Conclusion

This study shows that WES reanalysis in standard patient care leads to a substantial increase in diagnoses in children with NDD without causing unsolicited findings. The presence of dysmorphic features was associated with a higher diagnostic yield.

### Supplementary Information

Below is the link to the electronic supplementary material.

Supplementary file1 (DOCX 61 KB)

## Data Availability

The data that support the findings of this study are available from the corresponding author, SK, upon reasonable request.

## Abbreviations

CNV: Copy number variant
HPO: Human Phenotype Ontology
ID: Intellectual disability
LUMC: Leiden University Medical Centre
NDD: Neurodevelopmental disorder
NGS: Next-generation sequencing
TIQ: Total intelligence quotient
VUS: Variant of unknown significance
WES: Whole-exome sequencing

## Declarations

### Dual publication

The results/data/figures in this manuscript have not been published elsewhere, nor are they under consideration (from you or one of your contributing authors) by another publisher.
