# Supplementary material for: Reanalysis of whole-exome sequencing (WES) data of children with neurodevelopmental disorders in a standard patient care context
Source: Eur J Pediatr. 2023 Oct 27;183(1):345–55. doi: 10.1007/s00431-023-05279-4 (PMC10858114; doi:10.1007/s00431-023-05279-4)
Supplement: Supplementary file 1 — Supplementary file1 (DOCX 61 KB) [file 431_2023_5279_MOESM1_ESM.docx]

**Supplementary material**

***Supplementary material S1 Questionnaire send to academic centers*Questionnaire that was send to lab specialist:**

1. WES General:
a. How many WES analyses are performed in your center approximately each year?
b. What percentage of patients with developmental delay/intellectual disability/congenital disorders who undergo WES analysis receive a molecular diagnosis (diagnostic yield)?
c. How is the WES data analyzed (HPO terms/gene panel/open exome with filtering tool)? Has this approach changed in recent years?
d. Is CNV analysis also performed directly on WES data?

2. WES Reanalysis:
a. Is WES reanalysis performed in your center? When did you start doing this?
b. Does the reanalysis only involve reanalyzing the existing data, or is new data also generated?
i. Data reanalysis:
- Is the approach to data reanalysis (HPO terms/gene panel/open exome) different now than at initial analysis?
ii. Generating new data:
- When and for what reasons do you choose to generate new data?
- What sequencing technique is used?
- Is this technique different from the one used in the initial analyses?

3. Do you know the yield of WES data reanalysis (on existing and new data) for developmental delay/intellectual disability/congenital disorders in your center?
a. Would you be willing to share a percentage?
b. Are there any other results you would like to share?
c. Can this information be shared/used in a publication?

4. Is there anything else you would like to share about your experience with WES reanalysis?

**Questionnaire** **that was send to clinician:**

1. WES Reanalysis:
a. Who takes the initiative to request reanalysis of a previously negative WES?
b. For which indications do you request WES reanalysis?
c. How many years after the initial analysis is reanalysis usually performed?

2. Do you know the yield of WES data reanalysis (on existing and new data) for developmental delay/intellectual disability in your clinic?
a. Would you be willing to share a percentage?
b. Are there any other results you would like to share?
c. Can this information be shared/used for publication?

3. Is there anything else you would like to share about your experience with WES reanalysis?

***Supplementary material S2 Characteristics of initial analysis and reanalysis***

| Characteristics | Analyses (n=159) |
| --- | --- |
| Time between analyses (years mean ± SD) | 3.7 ± 1.2 |
| Outcome first analysis No possible explanatory variant Variant of uncertain significance Variant in candidate gene | 107 (67.3%) 29 (18.2%) 23 (14.5%) |
| Initiator of reanalysis Treating physician Parents/caretakes Clinical geneticist | 46 (28.9%) 10 (6.3%) 103 (64.7%) |
| Analysis method initial analysis Gene panel Moon analysis Gene panel and Moon analysis Gene panel and open exome analysis Moon and open exome analysis Gene panel, Moon and open exome | 24 (15.1%) 1 (0.6%) 5 (3.1%) 106 (66.7%) 2 (1.3%) 10 (6.3%) |
| Analysis method reanalysis Gene panel Moon analysis Gene panel and Moon analysis Gene panel and open exome analysis Moon and open exome analysis Gene panel, Moon and open exome | 1 (0.6%) 9 (5.7%) 13 (8.2%) 4 (2.5%) 121 (76.1%) 11 (6.9%) |
| Difference in analysis methods None Switch from gene panel and open exome to Moon and open exome analysis Add Moon analysis Switch to open exome (with or without changes in other methods) Drop gene panel only Switch from gene panel to Moon Drop open exome analysis (and gene panel) Add gene panel | 7 (4.4%) 91 (57.2%) 26 (16.4%) 12 (7.5%)  10 (6.3%) 5 (3.1%) 5 (3.1%) 3 (1.9%) |

***Supplementary material S3 Overview of new variants found with reanalysis***

| Patient ID | HPO Terms | First analysis* | Re-analysis* | Gene  OMIM number | Variant found at reanalysis | Variant type | Classification | Segregation** | Inheritance*** | Why found |
| --- | --- | --- | --- | --- | --- | --- | --- | --- | --- | --- |
| 5 | Cognitive impairment, Hearing impairment, Abnormality of toe, Obesity, Overfolded helix, Single transverse palmar crease | GP | GP  MA | *DCHS1*  *603057 | c.2425G>A  p.(Val809Met)  Homozygous | Missense | VUS in known gene | N/A | N/A | Gene panel update |
| 6 | Cognitive impairment, Autism, Muscular hypotonia, Laryngomalacia, Broad forehead, Downslanted palpebral fissures, Hemangioma, Periauricular skin pits, Broad jaw | GP OE | MA OE | *TMLHE*  *300777 | c.639-7C>G  p.(?)  Hemizygous | Splice-site | VUS in known gene | Maternal | N/A | Filtering update |
| 7 | Cognitive impairment, Esotropia, Ptosis, Epicanthus | GP OE | MA OE | *FOXP1*  * 605515 | c.1573C>T  p.(Arg525*)  Heterozygous | Nonsense | (Likely) Pathogenic | De novo | AD | Filtering update |
| 8 | Cognitive impairment, Macrocephaly, Speech delay, Motor delay | GP OE | MA OE | *RTN4* * 604475 | c.3536+4_3536+7del  p.?  Heterozygous | Splice-site | VUS in Candidate gene | De Novo | N/A | Filtering update |
| 24 | Cognitive impairment, Anteverted nares, Epicanthus, Hypermetropia, Strabismus, Overfolded helix, Sacral dimple, Neonatal hypotonia | GP OE | MA OE | *FOXP1*  * 605515 | c.1531-64_1531del p.(?)  Heterozygous | Splice-site | (Likely) Pathogenic | Paternal mosaicism | AD | Filtering update |
| 26 | Cognitive impairment, Epileptic spasm, Behavioral abnormality, Stapes ankylosis, Muscular hypotonia, Fatigue | GP OE | MA OE | *TSC2*  * 191092 | c.4863C>G  p.(Ile1621Met)  Heterozygous | Missense | VUS in known gene | Maternal | N/A | Moon Analysis |
| 27 | Cognitive impairment, Small for gestational age, Feeding difficulties, Behavioral abnormality, Abnormality of the dentition, Strabismus | GP OE | MA OE | *AGO1*  * 606228 | c.2465+1G>A  p.(?)  Heterozygous | Splice-site | VUS in known gene | Maternal | N/A | Moon Analysis |
| 30 | Cognitive impairment, Autism, Transposition of the great arteries, Behavioral abnormality | GP | MA OE | *ASH1L*  * 607999 | c.2029A>G  p.(Ser677Gly) Heterozygous | Missense | VUS in known gene | N/A | N/A | More knowledge about gene |
| 33 | Cognitive impairment, Failure to thrive, Microcephaly | GP | GP  MA | *BPTF*  *601819 | c.3233_3237del  p.(Arg1078Metfs*13) Heterozygous | Frameshift | (Likely) Pathogenic | N/A | AD | New gene discovery |
| 35 | Cognitive impairment, Joint hypermobility, Muscular hypotonia, Toe syndactyly, Epicanthus, Ptosis, Wide nasal bridge | OE | MA OE | *FBXO11*  *607871 | c.442G>A  p.(Ala148Thr)  Heterozygous | Splice-site | (Likely) Pathogenic | De Novo | AD | New gene discovery |
| 37 | Cognitive impairment, Tall stature, Speech delay, Mild myopia | GP | MA OE | *SIN3A*  *607776 | c.3259_3261del  p.(Glu1087del) Heterozygous | In-frame deletion | VUS in known gene | N/A | N/A | Interpretation error |
| 39 | Intellectual disability (mild), Gastrostomy tube feeding in infancy, Delayed speech and language development | GP OE | MA OE | *GRIN2B*  *138252 | c.1660T>A  p.(Phe554Ile)  Heterozygous | Missense | (Likely) Pathogenic | De Novo | AD | More knowledge about gene |
| 51 | Intellectual disability, Autism, Absent speech, Febrile seizures, Impaired toileting ability | GP OE | MA OE | *OTUD6B*  *612021 | c.929C>T  p.(Ser310Leu)  Homozygous | Missense | VUS in known gene | Biparental | AR | New gene discovery |
| 54 | Intellectual disability, Attention deficit hyperactivity disorder, Sleep disturbance, Obesity, Speech articulation difficult, Abnormal shape of the frontal, Brachycephaly, Abnormality of pain sensation | GP OE | MA OE | *PCDHGA3* *606290 | c.1123G>T  p.(Asp375Tyr) Heterozygous | Missense | VUS in candidate gene | De Novo | N/A | Changed reporting VUS |
| 57 | Intellectual disability (mild), Delayed speech and language development, Developmental regression, Downslanted palpebral fissure, Medial flaring of the eyebrow | GP OE | MA OE | *CACNA1C* *114205 | c.1969del  p.(Leu657Serfs*47) Heterozygous | Frameshift | VUS in candidate gene | De Novo | N/A | Changed reporting VUS |
| 59 | Intellectual disability (moderate), Delayed speech and language development, Delayed gross motor development | GP OE | MA OE | *AGPAT4* *614795 | c.872C>T  p.(Thr291Met) Homozygous | Missense | VUS in candidate gene | N/A | N/A | Changed reporting VUS |
| 72 | Intellectual disability, Polyneuropathy, Isomerism | GP OE | MA OE | *IRF2BPL* *611720 | c.1784dup  p.(Pro596Serfs*19)  Heterozygous | Frameshift | (Likely) Pathogenic | De Novo | AD | New gene discovery |
| 75 | Intellectual disability (mild), Precocious puberty, Microcephaly, Alopecia areata, Thoracic kyphosis, Short stature | GP OE | MA OE | *RAD21* *606462 | c.1074G>C  p.(Lys358Asn)  Heterozygous | Missense | VUS in known gene | Paternal | N/A | Moon Analysis |
| 91 | Global developmental delay, Joint hypermobility, Cataplexy, Speech apraxia, Highly arched eyebrow | GP OE | GP MA | *KCNMA1* *600150 | c.1606A>C  p.(Asn536His)  Heterozygous | Missense | (Likely) Pathogenic | De Novo | AD | New gene discovery |
| 93 | Intellectual disability, Celiac disease | GP OE | GP MA | *SETD5* *615743 | c.2347-7A>G  p.(?)  Heterozygous | Splice-site | (Likely) Pathogenic | De Novo | AD | Filtering update |
| 94 | Intellectual disability, Short stature, Congenital cataract, Rod-cone dystrophy, Cardiomyopathy, Hypospadias, Coarse hair, Oligodontia, Aplasia/Hypoplasia of the nails | GP OE | GP MA OE | *CWC27* *617170 | c.772_775del  p.(Val259Metfs*21)  Homozygous | Frameshift | (Likely) Pathogenic | Biparental | AR | New gene discovery |
| 98 | No HPO terms (no Moon), description phenotype: intellectual disability, cerebellar hypoplasia, hypotonia, ataxia, Erythema palmare, Failure to thrive, Scoliosis, hypermobility, swallowing difficulties | GP OE | GP OE | *CCT7* *605140 | c.377G>T  p.(Arg126Leu) Heterozygous | Missense | VUS in candidate gene | De Novo | N/A | Changed reporting VUS |
| 114 | Intellectual disability, Autism, Coarse facial features, Prominent eyelashes, Full cheeks, Synophrys | GP OE | MA OE | *SPEN* *613484 | c.2990dup  p.(Gln998Alafs*24)  Heterozygous | Frameshift | (Likely) Pathogenic | De Novo | AD | New gene discovery |
| 115 | Intellectual disability (mild), Encephalopathy, Ataxia, Nonprogressive visual loss, Absent speech, Motor delay, Seizures | GP OE | MA OE | *NPRL3* *600928 | c.1111_1122del  p.(Pro371_Leu374del)  Heterozygous | In-frame deletion | VUS in known gene | Paternal | N/A | Moon Analysis |
| 121 | Intellectual disability (mild) | GP | MA OE | *TAOK1* *610266 | c.2152A>T  p.(Lys718*)  Heterozygous | Nonsense | (Likely) Pathogenic | De Novo | AD | New gene discovery |
| 130 | Neurodevelopmental delay, Seizures, Muscle weakness, Hypertrophic cardiomyopathy, Abnormality of brain morphology, Partial agenesis of the corpus callosum, Congenital contracture, Dysphagia, Hypertonia | GP MA FU | MA OE | 1. *WDR41* *617502 2. *ZDHHC16* *616750 | 1. c.1101dup  p.(Asn368*) Homozygous  2. c.971+2T>C  p.? Homozygous | 1. Nonsense 2. Splice-site | VUS in candidate genes | N/A | N/A | Filtering update |
| 136 | Intellectual disability (moderate), Delayed speech and language development, Gray matter heterotopia, Abnormality of neuronal migration | GP MA FU | MA OE | *HDAC3* *605166 | c.277G>A  p.(Asp93Asn) Heterozygous | Missense | VUS in candidate gene | De Novo | N/A | More knowledge about gene |
| 137 | Intellectual disability, Basal cell carcinoma, Abnormal walk | GP MA | GP MA | *PTCH1* *601309 | c.3284_3298del p.(Glu1095_His1099del) Heterozygous | In-frame deletion | (Likely) Pathogenic | Mosaicism | AD | Change patient characteristics |
| 139 | No HPO terms (no Moon), description phenotype: intellectual disability, atrial septum defect, microcephaly, very small height, obesity, hearing loss and low vision. | GP | GP | *AFF4* *604417 | c.772C>T p.(Arg258Trp)  Heterozygous | Missense | (Likely) Pathogenic | Maternal mosaicism / De Novo | AD | Filtering update |
| 140 | Intellectual disability, Developmental delay, Short stature, Triangular face, Sparse hair, Eczema, Absence seizures, Anxiety disease | GP MA | GP MA | *ZMYM2* *602221 | c.3538C>T  p.(Arg1180*)  Heterozygous | Nonsense | (Likely) Pathogenic | N/A | AD | New gene discovery |
| 144 | Ataxia, Dysarthria, Delayed gross motor development, Global developmental delay |  | MA  OE | *SCN8A*  *600702  *KDM5B*  *605393 | c.272A>C p.(GLn91Pro)  Heterozygous  c.1853A>G p.(Tyr618Cys)  Heterozygous | Missense  Missense | VUS in known gene  VUS in known gene | De novo  De novo | AD  AR | Progression of phenotype |
| 145 | Motor delay, Short attention span, Hypotonia, Talipes equinovarus, Recurrent infections, Short stature, Oculomotor apraxia, Neutropenia, Fatigue | GP OE | MA OE | *EIF4H* *603431 | c.662C>T,  p.(Pro221Leu) Heterozygous | Missense | VUS in candidate gene | De Novo | N/A | Changed reporting VUS |
| 147 | Intellectual disability, Autistic behavior, Attention deficit-hyperactivi ty disorder, Behavioral problems, Bifid uvula | GP | MA | *SUZ12* *6006245 | c.1023+1G>C  p.(?)  Heterozygous | Splice-site | (Likely) Pathogenic | De Novo | AD | Gene panel update |
| 148 | Mild mental retardation, Global developmental delay, Motor delay, Poor speech, Language delay, Behavioral problems | GP OE | MA OE | *ZMYND8* *615713 | c.660G>T  p.(Lys220Asn) Heterozygous | Splice-site | VUS in candidate gene | N/A | N/A | Changed reporting VUS |
| 151 | Neurodevelopmental delay, Joint laxity, Muscle weakness, Hypotonia Hypertelorism, High forehead, Anomalous origin of left coronary artery from the pulmonary artery, Abnormality of cardiovascular system morphology | GP | MA | *SYT1* *185605 | c.1016A>G p.(Tyr339Cys)  Heterozygous | Missense | (Likely) Pathogenic | De Novo | AD | Gene panel update |
| 160 | Intellectual disability,  Developmental delay, Microcephaly,  Congenital cataract ,Hooked nose, Low frontal  hairline, Periventricular white matter hypodensities | MA  CP  OE | MA  OE | *ITSN1*  *602442 | c.3947G>C p.(Gly1316Ala)  Heterozygous | Missense | VUS in known gene | De novo | AD | More information about phenotype associated with gene |
| 164 | Intellectual disability (mild), Abnormal emotion/affect behaviour, Downslanted palpebral fissure, Bilateral ptosis, Cafe-au-lait spot | GP OE | MA OE | *TNRC6B* *610740 | c.4377del  p.(Lys1460Asnfs*56)  Heterozygous | Frameshift | VUS in known gene | Paternal | N/A | New gene discovery |
| 167 | Neonatal respiratory distress, Tachypnea, Generalized hypotonia, Motor delay; Language delay, Frontal bossing, Low nasal bridge, Anteverted nose, Autism | GP  OE | MA  OE | *GRIK2*  *611092 | c.1969G>A p.(Ala 657Thr) | Missense | VUS in known gene | De novo | AD |  |
| 171 | Delayed speech and language development, Short attention span, Microcephaly, Ventricular septal defect, Failure to thrive, Recurrent infections, Abnormality of the chin, Hypotelorism, Abnormality of the pinna, Conductive hearing impairment | GP OE | MA OE | *SPEN* *613484 | c.4441_4444del  p.(Glu1481Argfs*14) Heterozygous | Frameshift | (Likely) Pathogenic | De Novo | AD | New gene discovery |
| 177 | Intellectual disability, Autism spectrum disorder, Mental retardation (moderate), Cup-shaped ears, Malformed auricles | OE | MA OE | *POU3F3* *602480 | c.655_656del  p.(Leu220Alafs*419) Heterozygous | Frameshift | (Likely) Pathogenic | De Novo | AD | New gene discovery |
| 180 | Psychomotor retardation (moderate) | OE | MA OE | *SATB2* *608148 | c.1375C>T  p.(Arg459*)  Heterozygous | Nonsense | (Likely) Pathogenic | De Novo | AD | Gene panel update |

* GP = Gene panel, MA = Moon analysis, OE = Open Exome Analysis
** Segregation information not available if trio WES was not performed
*** Inheritance not known if pathogenicity of variant is not clear.

***Supplementary material S4 HPO terms used in more than 5% of the patients and their association with diagnostic yield.*** *This analysis is conducted within the group of patients for whom Moon Analysis was used for the reanalysis (n=154).*

| HPO Code | HPO Description | Frequency of term  (% of cases) | Diagnostic yield, % *Within with Within without*  *characteristic characteristic* | | P-value* |
| --- | --- | --- | --- | --- | --- |
| HP:0000750 | Delayed speech and language development | 45 (29.2) | 4.4 | 15.6 | 0.063 |
| HP:0000717 | Autism | 41 (26.6) | 4.9 | 15.0 | 0.104 |
| HP:0001256 | Intellectual disability, mild | 39 (25.3) | 7.7 | 13.9 | 0.405 |
| HP:0100543 | Cognitive impairment | 35 (22.7) | 11.4 | 12.6 | 1 |
| HP:0001249 | Intellectual disability | 32 (20.8) | 21.9 | 9.8 | 0.076 |
| HP:0001250 | Seizures | 27 (17.5) | 0.0 | 15.0 | 0.026 |
| HP:0001263 | Global developmental delay | 17 (11.0) | 5.9 | 13.1 | 0.696 |
| HP:0000729 | Autism spectrum Disorder | 14 (9.1) | 14.3 | 12.1 | 0.684 |
| HP:0000708 | Behavioral abnormality | 13 (8.4) | 15.4 | 12.1 | 0.664 |
| HP:0000252 | Microcephaly | 13 (8.4) | 15.4 | 12.1 | 0.664 |
| HP:0007018 | Attention deficit hyperactivity disorder | 12 (7.8) | 8.3 | 12.7 | 1 |
| HP:0001252 | Hypotonia | 11 (7.1) | 27.3 | 11.2 | 0.138 |
| HP:0001270 | Motor delay | 11 (7.1) | 0.0 | 13.3 | 0.361 |
| HP:0001344 | Absent speech | 9 (5.8) | 11.1 | 12.4 | 1 |
| HP:0000494 | Downslanted palpebral fissure | 9 (5.8) | 11.1 | 12.4 | 1 |
| HP:0000508 | Ptosis | 9 (5.8) | 22.2 | 11.7 | 0.307 |
| HP:0001508 | Failure to thrive | 8 (5.2) | 25.0 | 11.6 | 0.257 |
| HP:0002342 | Intellectual disability, moderate | 8 (5.2) | 12.5 | 12.3 | 1 |
| HP:0004322 | Short stature | 8 (5.2) | 12.5 | 12.3 | 1 |
| HP:0012758 | Neurodevelopmental delay | 8 (5.2) | 12.5 | 12.3 | 1 |

* p-value by Fisher exact test for the comparison of the group with and without the specific characteristic. After Bonferroni’s correction p-value < 0.003 was considered statistically significant.

***Supplementary material S5 HPO term phenotype groups and their association with diagnostic yield.*** *This analysis is conducted within the group of patients for whom Moon Analysis was used for the reanalysis (n=154).*

| Phenotype groups based on HPO terms | Number of patients in HPO group | Diagnostic yield, % *Within with characteristic* | *Within without characteristic* | p-value* |
| --- | --- | --- | --- | --- |
| Psychological/psychiatric disorder *- Autism* | 75 55 | 9.3 7.3 | 15.2 15.2 | 0.331 0.204 |
| Striking facial features | 59 | 13.6 | 11.6 | 0.802 |
| Structural neurological abnormality | 19 | 0.0 | 14.1 | 0.131 |
| Neurological disorder  *- Seizures* | 58 36 | 5.2 2.8 | 16.7 15.3 | 0.043 0.047 |
| Abnormal head circumference | 25 | 8.0 | 13.2 | 0.740 |
| Visual impairment / eye abnormality | 17 | 11.8 | 12.4 | 1 |
| Hearing impairment | 6 | 16.7 | 12.2 | 0.553 |
| Dermatologic condition | 8 | 12.5 | 12.3 | 1 |
| Cardiological malformation | 15 | 20.0 | 11.5 | 0.401 |
| > 5 HPO terms used | 52 | 10.3 | 13.5 | 0.622 |

* p-value by Fisher exact test for the comparison of the group with and without the specific characteristic. After Bonferroni’s correction p-value < 0.004 was considered statistically significant.
